# Reliable and Interpretable Mortality Prediction With Strong Foresight in COVID-19 Patients: An International Study From China and Germany

**DOI:** 10.3389/frai.2021.672050

**Published:** 2021-09-03

**Authors:** Tao Bai, Xue Zhu, Xiang Zhou, Denise Grathwohl, Pengshuo Yang, Yuguo Zha, Yu Jin, Hui Chong, Qingyang Yu, Nora Isberner, Dongke Wang, Lei Zhang, K. Martin Kortüm, Jun Song, Leo Rasche, Hermann Einsele, Kang Ning, Xiaohua Hou

**Affiliations:** ^1^ Division of Gastroenterology, Union Hospital, Tongji Medical College, Huazhong University of Science and Technology, Wuhan, China; ^2^ Key Laboratory of Molecular Biophysics of the Ministry of Education, Hubei Key Laboratory of Bioinformatics and Molecular-imaging, Department of Bioinformatics and Systems Biology, College of Life Science and Technology, Huazhong University of Science and Technology, Wuhan, China; ^3^ Department of Internal Medicine II, University Hospital of Würzburg, Würzburg, Germany

**Keywords:** COVID-19, Wuhan cohort, Würzburg cohort, mortality prediction model, reliability, interpretability, foresight

## Abstract

Cohort-independent robust mortality prediction model in patients with COVID-19 infection is not yet established. To build up a reliable, interpretable mortality prediction model with strong foresight, we have performed an international, bi-institutional study from China (Wuhan cohort, collected from January to March) and Germany (Würzburg cohort, collected from March to September). A Random Forest-based machine learning approach was applied to 1,352 patients from the Wuhan cohort, generating a mortality prediction model based on their clinical features. The results showed that five clinical features at admission, including lymphocyte (%), neutrophil count, C-reactive protein, lactate dehydrogenase, and α-hydroxybutyrate dehydrogenase, could be used for mortality prediction of COVID-19 patients with more than 91% accuracy and 99% AUC. Additionally, the time-series analysis revealed that the predictive model based on these clinical features is very robust over time when patients are in the hospital, indicating the strong association of these five clinical features with the progression of treatment as well. Moreover, for different preexisting diseases, this model also demonstrated high predictive power. Finally, the mortality prediction model has been applied to the independent Würzburg cohort, resulting in high prediction accuracy (with above 90% accuracy and 85% AUC) as well, indicating the robustness of the model in different cohorts. In summary, this study has established the mortality prediction model that allowed early classification of COVID-19 patients, not only at admission but also along the treatment timeline, not only cohort-independent but also highly interpretable. This model represents a valuable tool for triaging and optimizing the resources in COVID-19 patients.

## Introduction

The pandemic of coronavirus disease 2019 (COVID-19) has become a public health emergency of international concern (Salyer et al., 2021; Sirleaf and Clark, 2021; Watson and Lilford, 2021). As of July 12, 2021, 187, 796, 841 confirmed infection cases have been reported by the World Health Organization, with a global mortality rate of 2.16% (https://covid19.who.int/). Even worse, the incidence of COVID-19 is continuously increasing worldwide, and areas already under control are likely to relapse (Setti et al., 2020). The proportion of critically ill COVID-19 patients is 18.5% (Epidemiology Working Group for Ncip Epidemic Response CCfDC, Prevention, 2020), and this high proportion of severe cases has put enormous pressure on medical systems, resulting in a serious shortage of medical resources (Rasmussen et al., 2020; Ammar et al., 2021; Wahlster et al., 2021).

In recent years, machine learning methods used for large clinical data analysis have been sprung up (Liang et al., 2020; Wu et al., 2020; Xiao et al., 2020; Zhu et al., 2020; Gomes and Serra, 2021; Ikemura et al., 2021; Wang et al., 2021). Yan et al*.* used the XGBoost classifier (Chen and Guestrin, 2016) to predict the outcome of 485 patients using the final samples at discharge, and they found three blood features that could be used as predictors, providing important evidence for clinical decision-making and patient management (Liang et al., 2020). Xiao et al*.* have used the HNC-LL score that considered hypertension, neutrophil count, C-reactive protein (CRP), lymphocyte count, and lactate dehydrogenase (LDH) to predict the severity of COVID-19 with AUC higher than 0.82 based on 442 patients (Xiao et al., 2020). Liang et al. developed a deep learning survival Cox model for 1,590 patients’ triage, which was based on four clinical features and six phenotypic characteristics, to ensure patients at the greatest risk for severe illness receive appropriate care as early as possible (Liang et al., 2020). Wu et al*.* also used the Cox model to investigate the key risk factors and predicted the mortality rate of 21,392 COVID-19 patients based on demographic, clinical, and laboratory features and found that the mortality rate increased with time, especially for these critically ill patients (Wu et al., 2020).

Unfortunately, although the clinical features of COVID-19 patients have been reported in several recent publications (Gupta et al., 2020a; Xu et al., 2020), such as decreased lymphocytes and elevated CRP (Gupta et al., 2020a; Xu et al., 2020), the predictive powers and interpretations of these clinical features remain unclear. Additionally, since progression and outcome are critical for COVID-19 patients (Liang et al., 2020; Risch, 2020), timely monitoring from admission to outcome also has important clinical significance, making it possible to adjust treatment regimens in time, but this process is not entirely clear. Moreover, the foresight of a predictive model, as to how many days before discharge these features could accurately predict the patients’ outcome, remains elusive. However, the association of these clinical features with phenotypic characteristics is also unclear. The robustness of the mortality prediction model along the timeline and the predictive power considering different preexisting diseases also need further exploration. Therefore, we performed this international, bi-institutional study to establish a mortality prediction model with the aim of early triaging and optimizing the resources.

## Methods

### Ethical Approval

This study was approved by the Ethics Committee of Union Hospital, Tongji Medical College, Huazhong University of Science and Technology. Due to the retrospective nature of this study, the local institutional review board of the University of Würzburg waived the requirement for additional approval. This study was performed in accordance with the ethical standards laid down in the 1964 Declaration of Helsinki and its later amendments.

### Sample Description

Clinical data were collected from 1,441 COVID-19 patients from January 28, 2020, to March 29, 2020, at Wuhan Union Hospital (also called Wuhan cohort), China, for model development. Moreover, 96 patients with confirmed COVID-19 disease were collected from the University Hospital of Würzburg (also called Würzburg cohort), Germany, from March 6, 2020, to September 14, 2020, for independent test.

**FIGURE 1:**
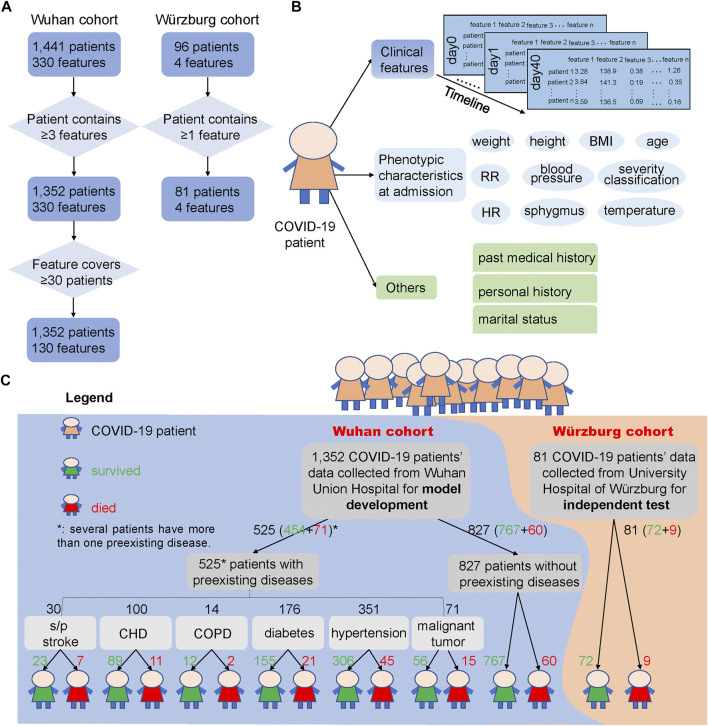
COVID-19 patients and their clinical feature filtering process, phenotypic characteristics and clinical features used in this study, and the outcome of the two cohorts. **(A)** Process of filtering low-quality samples of the two cohorts. Here, 330 features in the Wuhan cohort were the union of 1,441 patients’ clinical features and 130 features were the union of the 1,352 patients’ clinical features after filtering. “≥3 features” says that the patients from the Wuhan cohort should contain at least three clinical features during the hospital stays, and “≥30 patients” says that the clinical features that collected from clinical laboratory should cover at least 30 patients and thus could be used for subsequent analysis. In the Würzburg cohort, “≥1 feature” says that the patient should contain at least one of the features from these four clinical features: lymphocyte (%), neutrophil count, LDH, and CRP. **(B)** Different types of clinical features and phenotypic characteristics used in the two cohorts. We used clinical features from hospital laboratory tests for developing the prediction model, and these clinical features were also used to test the association with phenotypic characteristics and other records. **(C)** Overview of samples used for model development and independent test. Samples of 1,352 COVID-19 patients from Wuhan Union Hospital (Wuhan cohort, the blue background) were used for building and testing the mortality prediction model, while samples of 81 COVID-19 patients from Germany (Würzburg cohort, the orange background) were used for independent test of the mortality prediction model. The green number represents the number of patients who survived from COVID-19, while the red number means the number of patients who died from COVID-19. Note that several patients have more than one preexisting disease.

For the Wuhan cohort, more than 300 clinical features from hospital laboratory tests were recorded, and most patients have multiple sets of clinical features during their stay in the hospital. In addition, physical examinations, such as height, weight, temperature, sphygmus, systolic/diastolic pressure, respiratory rate, and heart rate, were performed upon admission of these COVID-19 patients. For robust analysis, clinical features that covered less than 30 samples, as well as samples containing fewer than three clinical features, were discarded (Figure 1A). After filtering out low-quality records, 1,352 patients and 130 clinical features were selected for systematic analysis. The average age of these patients was 58.22 (standard error: 14.90), and 50.52% of them were male, indicating a balanced gender. The minimal, maximal, and median duration from admission to discharge of the 1,352 patients is 0, 55, and 10 days, respectively. Among all of 1,352 COVID-19 patients, 1,221 patients survived and 131 died (Supplementary Table S1).

Clinical features (Figure 1B) from hospital laboratory tests were primarily composed of two parts: 101 numerical features, such as LDH and CRP, and 29 binary features, such as ABO blood type, Mp-IgM, and Mp-IgG. These clinical features were considered as candidate biomarkers for COVID19 mortality prognosis.

Phenotypic characteristics at admission (Figure 1B) were primarily composed of two parts: numerical and binary phenotypic characteristics. The numerical phenotypic characteristics included age, height, weight, temperature, sphygmus, systolic/diastolic pressure, respiratory rate, heart rate, and clinical classification. Binary phenotypic characteristics included records of gender, smoking status, and blood type.

Recent studies have already reported that the outcome of COVID-19 patients is greatly influenced by whether the patient has a preexisting disease (Azevedo et al., 2020; Zhou et al., 2020a; Williamson et al., 2020), such as CHD (Mai et al., 2020), hypertension (Itoh, 2020), and diabetes (Gupta et al., 2020b). Here, we divided 1,352 COVID-19 patients into seven groups: s/p stroke (23 survived and seven died), CHD (89, 11), chronic obstructive pulmonary disease (COPD) (12, 2), diabetes (155, 21), hypertension (306, 45), malignant tumor (56, 15), and those without preexisting diseases (767, 60) according to their past medical history (Figure 1C).

For the 96 patients in the Würzburg cohort, we have filtered out the patient who has not a single clinical feature among the four clinical features (lymphocyte (%), neutrophil count, LDH, and CRP) (Figure 1A). After this process, 81 samples were retained and utilized for independent test. For these 81 patients, their phenotypic characteristics including systolic pressure, diastolic pressure, temperature, heart rate, SpO_2_, age, and respiratory rate were also used for analysis. The average age of these patients was 67.15 years (standard error: 15.17), which was significantly higher than that of patients in the Wuhan cohort (*t*-test, *p* = 0.0005). 62.96% of them are male, 53.67% of them have respiratory failure, and 41.46% of them need mechanical ventilation. Among them, 72 survived and nine died from COVID-19 (Supplementary Table S2).

### Severity Classification

According to the diagnosis and treatment of pneumonia infected by the new novel coronavirus (the trial seventh edition) (National Health Commission of the People’s Republic of China, 2020), the patient’s severity classification was divided into three classifications, general, severe, and critical, according to their symptoms at admission. In this work, among 1,352 patients from the Wuhan cohort, 896 were in general, 393 were in severe, and 63 were in critical. For the Würzburg cohort, 24 were in general, 35 were in severe, and 22 were in critical. Here, we defined severity classification as follows: general as 1, severe as 2, and critical as 3.

### Clinical Feature Profiling

Using patient samples at admission, all numerical clinical features were normalized to a range [0, 1]. These normalized data with an average abundance ≥0.001 were illustrated as boxplots using the R package “ggplot2”. To illustrate differences between patients who survived and died, as well as between patients with or without preexisting diseases, principal coordinate analysis (PCoA) was performed using all patients’ numerical clinical features at admission based on the Jaccard coefficient for distance measurement using the R package “vegan”.

### Feature Selection and Development of a Prediction Model Utilizing Clinical Features

To identify the most important clinical features that reflect differences among the samples, feature selection was employed for a deeper understanding of COVID-19 infection. We assessed the contribution of each clinical feature to facilitate the decisions of the algorithm. Considering both MeanDecreaseAccuracy and MeanDecreaseGini, the top five discriminatory clinical features were selected. Different Random Forest (RF) models were tested on the top five important clinical features, as well as their different combinations according to their importance.

To develop a mortality prediction model that is capable of distinguishing the outcome of COVID-19 patients, RF analysis was performed by randomForest() function in R (package “randomForest”). For the sample size larger than 100, we randomly selected 90% of samples as training set and 10% of samples as testing set using sample() function with replacement. In this process, replace parameter was set as true, which specifies using the Bootstrap method for random sampling. For each model, based on each training set, the important parameters ntree (number of decision trees contained in the RF model) and mtry (variable sampling values for each iteration) were trained and estimated with the out-of-bag (OOB) value. The importance was set as true for calculating the importance of each variable in the model, which was mainly used in conjunction with the importance() function. The proximity parameters were set as true for calculating the proximity matrix of the model, which is mainly used in conjunction with the MDSplot() function to realize the visualization of random forest. The na.action parameter specifies the methods for handling the missing values and was set as na.omi (that is, delete the samples with missing values of all features). Other parameters were set as default. A traversal search was performed on all clinical features to obtain the minimum OOB value. The value of mtry was determined by the OOB value (that is, the index of the minimum OOB value). Then, combining the outcome of COVID-19 patients, the mtry value was iterated to obtain an optimal ntree. This process was iterated 15,000 times or more to construct the most accurate model. When the error tree approaches stable, the minimum number of trees was the best value for ntree. This trained model was used for predicting the outcome of the testing set.

### Evaluation of Prediction Models

To evaluate the performance of the RF model, we used several standard statistic parameters: accuracy, precision, sensitivity or recall, specificity, and F1 scores. Here, we defined the prediction result: survived-survived as TP and died-died as TN. The formulas of the parameters mentioned above are defined as follows:

accuracy = (TP + TN)/(TP + TN + FP + FN), (1)

precision = TP/(TP + FP), (2)

recall = TP/(TP + FN), (3)

specificity = TN/(TN + FP), (4)

F1= 2* precision*recall/(precision + recall), (5)

where TP, TN, FP, and FN stand for true-positive, true-negative, false-positive, and false-negative rates, respectively.

### Correlation Analysis Between Phenotypic Characteristics and Clinical Features

To better understand the relationship between phenotypic characteristics and the mortality rate of patients, we used the Pearson coefficient to examine the correlation between phenotypic characteristics and clinical features. Again, we organized these correlation values along the timeline to identify the dynamics of such correlations during treatment progression.

### Evaluation of Prediction Models along the Timeline

Most patients have multiple sets of clinical features during their stay in the hospital, allowing for a series of mortality prediction models along the timeline. Here, we ordered these mortality prediction models in two directions: from admission forward to outcome to directly provide day-to-day guidance for clinics and from outcome backward to admission to evaluate the robustness and prediction power of the model against the time of hospital stay.

### Development of High-Quality and Interpretable Binary Decision Tree for Clinical Diagnosis

Based on the five selected features, we aimed to develop a high-quality decision tree for clinical diagnosis. To train the RF model, the dataset was randomly separated into two groups: the training set (90% of entries) was applied to construct the mortality prediction model, and the testing set (10% of entries) was applied to validate the mortality prediction model. For datasets with a sample size of less than 100, we used 70% of the dataset for training and 30% for testing to reduce the contingency error. This process was iterated 15,000 times to construct the most accurate model. The most discriminative clinical feature was used as the root node of this binary decision tree, and the child nodes were hierarchically formed according to their distinguishing ability until all samples are completely distinguished. Finally, the decision tree was visualized by rpart() function in R (package “party”).

### Development of a Prediction Model for Different Preexisting Diseases

Considering the influence of preexisting diseases on the outcome in COVID-19 patients, we also used the first samples of patients with preexisting diseases as a training dataset to build the mortality prediction models: s/p stroke, CHD, COPD, diabetes, hypertension, malignant tumor, and those without preexisting diseases. For a dataset with a sample size larger than 100, we used 90% of the dataset for training and 10% for validation. For a dataset with a sample size smaller than 100, we used 70% of the dataset for training and 30% for testing to validate the model to reduce the contingency error.

### Independent Test of the Mortality Prediction Model Using the Würzburg Cohort

To examine the reliability, interpretability, and foresight of our mortality prediction model developed based on the Wuhan cohort, 81 samples at admission from the Würzburg cohort were used for independent test. Pearson coefficient was also used to evaluate the association between the four clinical features (lymphocyte (%), neutrophil count, LDH, and CRP) and phenotypic characteristics (systolic pressure, diastolic pressure, temperature, heart rate, SpO_2_, age, and respiratory rate).

## Results

In this study, we have recruited two independent cohorts from China (the Wuhan cohort) and Germany (the Würzburg cohort) for building and testing a mortality prediction model, respectively. The Wuhan cohort contained 1,352 COVID-19 patients from Wuhan Union Hospital, and it has been utilized for establishing a multi-feature and time-series aware machine learning models. The Würzburg cohort consists of 81 COVID-19 patients and has been used as an independent validation cohort.

### Data Resource and General Profiles of COVID-19 Patients from Wuhan Cohort

1,352 patients were enrolled in the Wuhan cohort, who had more than three clinical features (such as neutrophil count, CRP, lymphocyte count, LDH, albumin, direct bilirubin, and creatine kinase) (Liang et al., 2020; Xiao et al., 2020) and detailed medicinal records from January 28, 2020, to March 29, 2020. The distribution of the number of patients with clinical laboratory tests on a daily basis, as well as the total number of diagnoses for each patient, is shown in Supplementary Figure S1. Among them, the mortality rates in patients with preexisting diseases: s/p stroke, coronary heart disease (CHD), chronic obstructive pulmonary disease (COPD), diabetes, hypertension, and malignant tumor were 23.33, 11.00, 14.29, 11.93, 12.82, and 21.13%, respectively (Supplementary Figures S2A,B). These mortality rates were significantly higher (*t*-test, *p* < 0.001) than those in patients without preexisting diseases (mortality rate: 7.26%). PCoA showed that if we used all clinical features, these patients cannot be clearly separated (Supplementary Figure S2C). In addition, these patients could not be separated by whether they had a preexisting disease or not (Supplementary Figures S2D–J). This highlights the importance of clinical feature selection and developing the mortality prediction models to differentiate patients.

### Development and Evaluation of Clinical Feature Selection and Mortality Prediction Model for Early Prognosis Based on Wuhan Cohort

We first developed a mortality prediction model based on patients’ samples at admission, since such prediction is of paramount importance in clinics (Risch, 2020). This model took the clinical features and outcomes into consideration, aiming to optimize the medical resources, as well as preemptive therapy.

Before developing a mortality prediction model, we divided the 130 clinical features into two parts: 101 numerical features and 29 binary features. For numerical features, those features which are identified with the average abundance ≥0.001 are shown in Supplementary Figure S3. 101 numerical clinical features with at least 30 samples’ coverage were considered as the outcome predictors and were used to build the mortality prediction model. We used 90% of the samples for model training and 10% for testing to validate the model.

**FIGURE 2:**
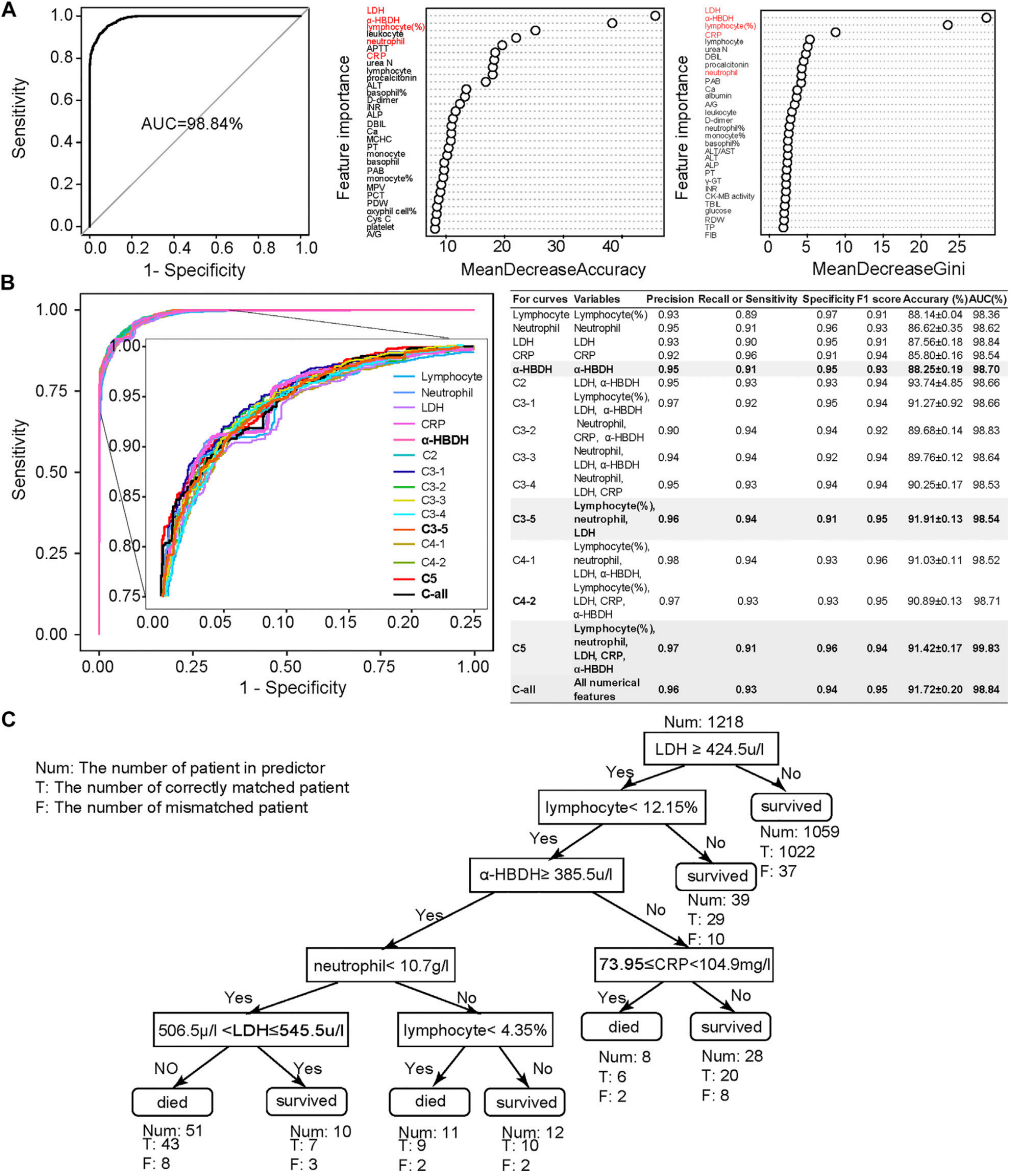
Clinical feature selection and mortality prediction results utilizing the first samples at admission. **(A)** Clinical feature selection based on 1,352 samples and 101 numerical features. Features were ranked by MeanDecreasedAccuracy and MeanDecreasedGini according to their importance. Considering both of these import RF parameters, we selected five important clinical features: lymphocyte (%), neutrophil count, LDH, CRP, and α-HBDH. **(B)** Comparison of receiver operating characteristic (ROC) curves and diagnostic performance of all numerical clinical features, subfeature combinations, as well as each selected single feature, using the first samples at admission (also referred to as admission-day 0). **(C)** The binary decision tree for predicting the outcome of COVID-19 patients based on the five selected clinical features at admission (admission-day 0). Annotations: Num: the number of patients in the predictor; T: the number of correctly matched patients; F: the number of mismatched patients. Here, the Num above the root node indicates the total samples used for building the binary decision tree.

Combined MeanDecreaseAccuracy and MeanDecreaseGini (Figure 2A), lymphocyte (%), neutrophil count, C-reactive protein (CRP), lactic acid dehydrogenase (LDH), and α-hydroxybutyric dehydrogenase (α-HBDH) were selected for developing an optimized model, where lymphocyte (%) is an immune disorder indicator (Trowell, 1947), neutrophil count represents infection (Xie et al., 2020), CRP represents inflammatory response (Vermeire et al., 2004; Sabrina et al., 2012), and both LDH and α-HBDH represent tissue lesions (Sanwald and Kirk, 1966; Kishaba et al., 2014).

We then used these five selected numerical clinical features (lymphocyte (%), neutrophil count, CRP, LDH, and α-HBDH), as well as different combinations of the subset of these five clinical features according to their importance, for prediction (Figure 2B). Results showed that the performance of these five clinical features could be comparable to the results predicted by all numerical features. Considering the F1 score, accuracy, and AUC, the combination of lymphocyte (%), neutrophil count, and LDH also showed high performance, especially the performance of α-HBDH used alone (bold in Figure 2B). Several specified combinations of three out of these five clinical features, such as the combination of lymphocyte (%), neutrophil count, and LDH, also reached more than 91% accuracy and 99% AUC at admission. However, in clinics, these five features covered more types of clinical symptoms: lymphocyte (%) is an immune disorder indicator (Trowell, 1947), neutrophil count represents infection (Xie et al., 2020), CRP represents inflammatory response (Vermeire et al., 2004; Sabrina et al., 2012), and both LDH and α-HBDH represent tissue lesions (Sanwald and Kirk, 1966; Kishaba et al., 2014). Thus, we confirmed these five clinical features as credible biomarkers.

To benchmark with other classification algorithms, we also used FEAST (an expectation–maximization-based unsupervised learning method) (Shenhav et al., 2019) and JSD (Jensen–Shannon divergence) methods (Lin, 1991) to predict the outcome of COVID-19 patients based on all features, the top five features, and the top three features (Supplementary Figure S4). Results demonstrated that the RF model was more or equally credible for constructing the mortality prediction model. The neural network (Kriegeskorte and Golan, 2019) with two hidden layers (the first layer has 128 neurons and the second layer has eight neurons) also illustrated that RF model based on the combination of lymphocyte (%), neutrophil, LDH, CRP, and α-HBDH could best predict the outcome of COVID-19 patients (Supplementary Figure S5). Moreover, all three methods (RF, FEAST, JSD, and neural network) showed the best distinguishing power when using the top five clinical features to construct the model.

We also used the binary clinical features (such as urine occult blood, blood type, and COVID-19 nucleic acid) to build the mortality prediction model (Supplementary Figure S6A). Based on the contribution of each feature, we selected urine protein (UPRO), urine occult blood (UOB), monospecific antibodies of blood type (Ab-monospecific-B), ABO blood type (ABO), and ketones (KET) for further model improvement. Their different combinations and performance are shown in Supplementary Figure S6B. Among them, the combination of UPRO, UOB, and KET (accuracy = 99.61%; AUC = 99.96%) was outstanding from the others, followed by UPRO, all binary features, and the combination of these five features.

Finally, we emphasized that all of the above results were based on the first samples at admission, since it is more important for the clinical prediction to utilize these samples. It was noticed that a recently published study used the final samples of COVID-19 patients for predicting their outcome (Yan et al., 2020), and we also used the final samples in the Wuhan cohort to assess our model based on five selected features (Supplementary Figure S7), with results showing high prediction accuracy. Yet, the prediction accuracy and AUC based on first samples at admission (Figure 2B) were comparable to those based on these final samples for the Wuhan cohort. These results confirmed again that patients with a high mortality rate could be accurately predicted at admission, which could be used for prioritizing critically ill patients to potentially reduce the mortality rate.

### Clinical Features Have Profound Association with Phenotypic Characteristics in the Wuhan Cohort

**FIGURE 3:**
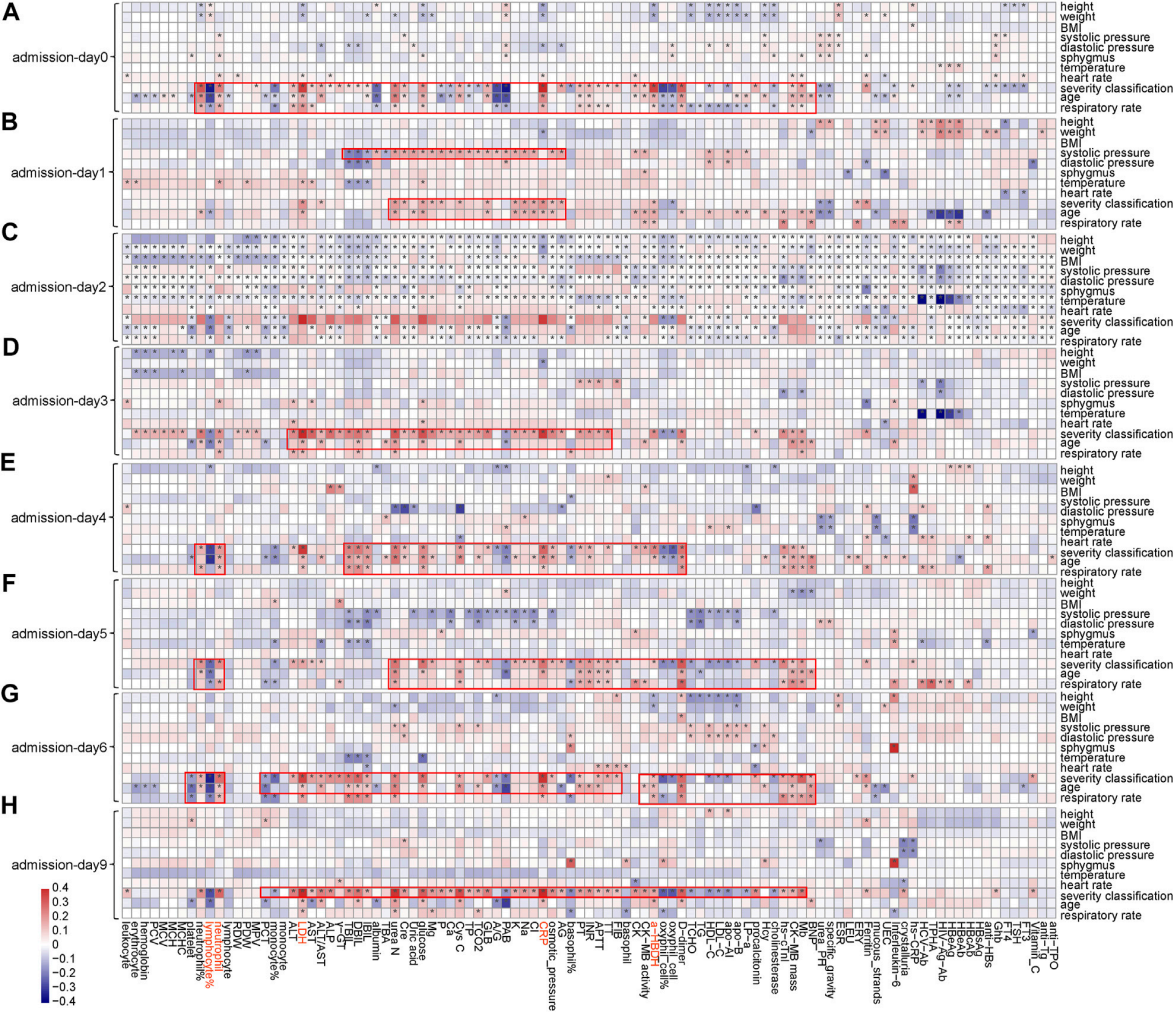
Associations between clinical features and phenotypic characteristics based on several representative time points. **(A)** Based on admission day (admission-day 0). **(B)** Based on the third day after admission (admission-day 1). **(C)** Based on the sixth day after admission (admission-day 2). **(D)** Based on the ninth day after admission (admission-day 3). **(E)** Based on the admission day (admission-day 4). **(F)** Based on the third day after admission (admission-day 5). **(G)** Based on the sixth day after admission (admission-day 6). **(H)** Based on the ninth day after admission (admission-day 9). Note: *represents a significant correlation between a phenotypic characteristic and a clinical feature (Pearson correlation: *p* < 0.05).

Notable correlations were observed between phenotypic characteristics and clinical features associated with COVID-19 (Figure 3 and Supplementary Figures S8, S9). Among 101 numerical clinical features, many of them have shown significant correlations with age, respiratory rate, and severity classification of patients. Expect for lymphocyte (%), neutrophil count, LDH, CRP, and α-HBDH were positively correlated with age (*p* < 0.05) along the timeline. Since the above analyses also confirmed that these five clinical features are tightly associated with patient outcomes (Figure 2), these associations partially verified the fact that elder patients were more likely to die from COVID-19. LDH, CRP, and α-HBDH were also positively correlated with respiratory rate and severity classification (*p* < 0.05) in patients (896 were in general, 392 were in severe, and 63 were in critical), illustrating the importance of these phenotypic characteristics on outcome in COVID-19 patients. The result also showed dynamic changes in the associations of these clinical features with phenotypic characteristics over time, especially for the five clinical features used for model prediction.

### Time-Series Analysis Reveals That the Mortality Prediction Model Is Very Robust along the Timeline

**FIGURE 4:**
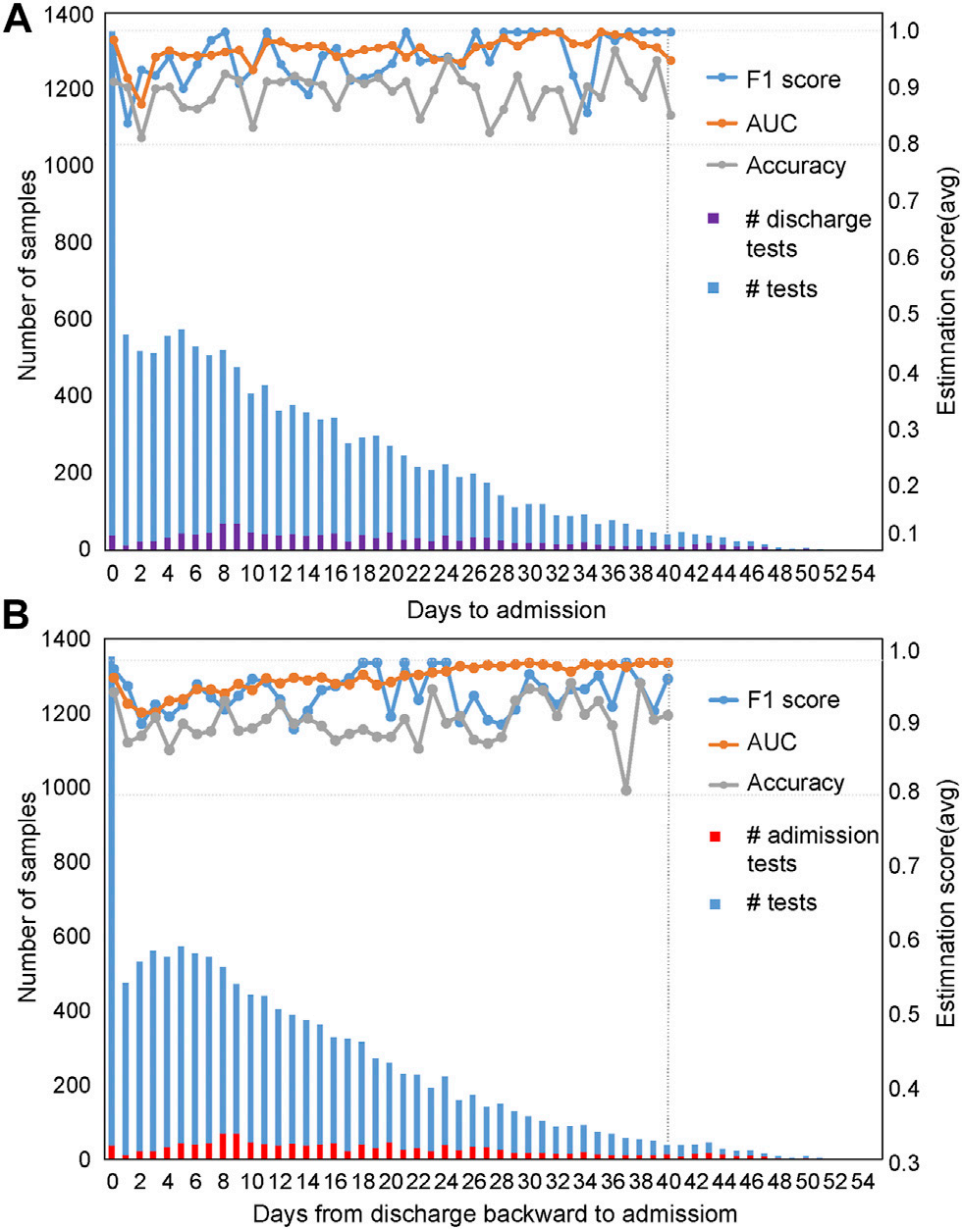
Evaluation of prediction results based on time series with five selected features. The prediction results were evaluated based on time series using the admission-day 0 **(A)** and the discharge-day 0 **(B)** as start points. The first *y*-axis represents the number of samples enrolled, while the second *y*-axis represents the estimation scores. The purple bar represents the number of patients discharged from the hospital on that day, while the red bar indicates the number of patients admitted to the hospital on that day.

***Evaluation of the mortality prediction model along the timeline forward from admission day as the start point:*** Because these clinical features are dynamic along the timeline, and in clinics, the progression and outcome of patients are critical (Liang et al., 2020; Risch, 2020). Therefore, we used the admission day of each patient as the start point and built mortality prediction models day by day after admission along the timeline. The number of samples enrolled on a daily basis is shown in Figure 4A from admission-day 0. We used 90% of the dataset for training and 10% for testing. For datasets with a sample size of less than 100, we used 70% of the dataset for training and 30% as a test set for validation. Since the sample number was less than 50 for patients who stayed in the hospital longer than 40 days, we only used the dataset from admission-day 0 to admission-day 40 to build the time-series mortality prediction models. Results confirmed that our mortality prediction model was very robust over time, suggesting that according to the prediction outcome of patients, clinics could adjust the treatment plan at any time, which could provide higher quality treatment for patients.

***Evaluation of the mortality prediction model along the timeline backward from discharge day as the start point:*** To prove the robustness of our mortality prediction model and how many days in advance it could predict the outcome of COVID-19 patients, we used the discharge day of each patient as the start point. Prediction accuracies were evaluated backward day by day (Figure 4B) from discharge-day 0. The mortality prediction model based on five clinical features also reached more than 91% accuracy and 99% AUC (usually 10 days or more in advance of the outcome) (Figure 4B), confirming this mortality prediction model is very robust over time when patients were in the hospital and indicating the strong association of these five clinical features with the progression of treatment.

### The Highly Accurate and Interpretable Binary Decision Tree for Clinical Diagnosis

To make the prediction interpretable, we also generated a series of decision trees (along the timeline) for assisting clinical diagnosis based on the Wuhan cohort. The decision tree is hierarchically organized by the distinguishing ability of these five clinical features based on the first samples at admission (Figure 2C). LDH could distinguish 87% of samples with more than 96% accuracy and was used as the root node of this decision tree. The remaining 13% of samples were differentiated by a combination of these five clinical features. The binary decision tree of the final samples at discharge was simpler than that of the first samples at admission (Supplementary Figure S10E). The decision trees based on other time points are shown in Supplementary Figures S10A–D, confirming that using these five clinical features was more comprehensive and precise. These results also suggested that the mortality prediction model based on the admission samples, rather than the discharge samples, could already provide outcome prediction and clinical guidance for personalized treatment with high fidelity.

The binary decision tree, either based on samples at admission or based on discharge, was also highly interpretable for clinical diagnosis. The elevated LDH was associated with patients’ death: LDH larger than 445 u/l was a significant risk factor related to death in cases with severe COVID-19 (Zhou et al., 2020b; Li et al., 2020), which was consistent with our results. The increased level of α-HBDH was also found as a critical risk factor associated with the severity of COVID-19 patients (Dong et al., 2020). The decreased amount of lymphocyte (lymphopenia) and neutrophil (neutrophilia), together with the increased number of CRP and LDH, showed the immunological response to the virus, followed by severe virus infection (Frater et al., 2020; Lippi and Plebani, 2020). In summary, current published clinical evidence could well support our decision tree.

### Prediction Power Considering Different Preexisting Diseases

For different preexisting diseases, the clinical features that can accurately mark the COVID-19 patients’ outcomes are generally different. Previous studies have shown that preexisting disease increases the risk of COVID-19 mortality rate (Williamson et al., 2020). We also used the six preexisting diseases to evaluate the mortality prediction model based on the five selected clinical features (Supplementary Figure S11).

Out of the five selected clinical features, feature combinations should be different for each of the different preexisting diseases. Therefore, for each of the preexisting diseases, we performed feature importance evaluation before mortality prediction model evaluation. For patients with s/p stroke (Supplementary Figure S11A), considering F1 score, accuracy, and AUC, the combination of LDH, CRP, and α-HBDH showed the highest performance, followed by the combination of LDH and α-HBDH, then all five features. The results for patients with CHD are illustrated in Supplementary Figure S11B. Except for using the five features, the combination of LDH, CRP, and α-HBDH showed the highest performance. For patients with COPD, a combination of neutrophil count, lymphocyte (%), and LDH showed the highest performance (Supplementary Figure S11C). For patients with diabetes, among all combinations of clinical features, LDH showed the highest performance (Supplementary Figure S11D), indicating that LDH could be used to distinguish the outcome of COVID-19 patients. For patients with hypertension, results indicated that a combination of neutrophil count, lymphocyte (%), and LDH could be used as biomarkers for predicting the outcome of COVID-19 patients with hypertension (Supplementary Figure S11E). For patients with malignant tumor, the combination of all five features showed the highest performance, followed by the combination of neutrophil count and lymphocyte (%) (Supplementary Figure S11F). For patients without preexisting diseases, results showed that we can use lymphocyte (%), LDH, CRP, and α-HBDH to accurately predict the outcome of these patients (Supplementary Figure S11G).

### Evaluation of the Mortality Prediction Model Using the Independent Würzburg Cohort

**FIGURE 5:**
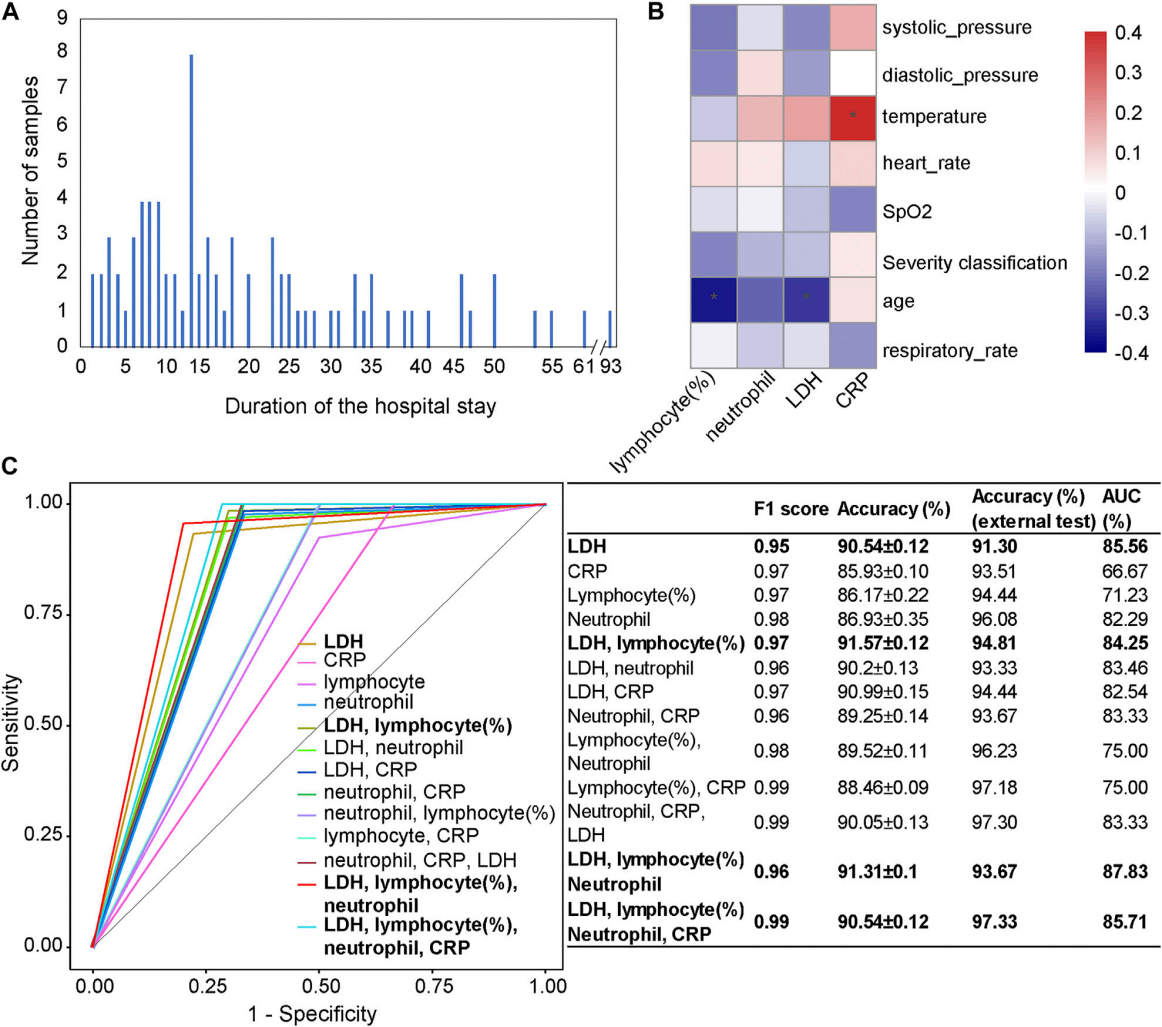
Independent test results on the Würzburg cohort utilizing the first samples at admission. **(A)** The number of patients categorized by their duration of the hospital stay. **(B)** The associations between clinical features (LDH, CRP, lymphocyte (%), and neutrophil count) and phenotypic characteristics. **(C)** Comparison of receiver operating characteristic (ROC) curves and diagnostic performance of four clinical features, the combinations of a subset of features, as well as each selected single feature, using the first samples at admission. Note: * represents a significant correlation between a phenotypic characteristic and a clinical feature (Pearson correlation: *p* < 0.05).

The reliability, interpretability, and foresight of our mortality prediction model were further confirmed in another independent cohort collected from Germany, the Würzburg cohort, with samples collected from March to September 2020 (Figure 5). For the patients in the Würzburg cohort, their duration of stay in the hospital is usually 5–20 days (Figure 5A). All samples used in the Würzburg cohort were the patient samples at admission.

We used four clinical features (lymphocyte (%), neutrophil count, LDH, and CRP), as well as their different combinations to test our mortality prediction model (Figure 5C). Considering F1 score, accuracy, and AUC, the combination of LDH, lymphocyte (%), neutrophil count, and CRP (accuracy = 97.33%; AUC = 85.71%) showed the highest performance among different combinations. Other combinations, such as the combination of LDH, lymphocyte (%), and neutrophil count (accuracy = 93.67%, AUC = 87.83%) and the combination of LDH and lymphocyte (%) (accuracy = 94.81%, AUC = 84.25%), also performed well. When only one clinical feature was used, LDH (accuracy = 91.30%, AUC = 85.56%) showed the highest performance, which was consistent with the results on the Wuhan cohort and a previous study (Liang et al., 2020).

From the Pearson correlation analysis (Figure 5B) between these four clinical features (LDH, lymphocyte (%), neutrophil count, and CRP) and the phenotypic characteristics (systolic pressure, diastolic pressure, temperature, heart rate, SpO_2_, age, and respiratory rate), we could observe that there was a significantly negative correlation between lymphocyte (%) and age (*p* < 0.05), which was consistent with the general pattern of COVID-19 patients. CRP was significantly positively correlated with temperature (*p* < 0.05), which was consistent with the result in the Wuhan cohort.

Furthermore, as the duration of stay in the hospital of patients is usually 5–20 days, the strong foresight of the mortality prediction model has again been validated on the Würzburg cohort. Furthermore, one male patient aged 54 has a hospital stay of 93 days before recovery, and our mortality prediction model has successfully predicted his outcome.

## Discussions and Conclusion

Our study enrolled two independent cohorts of COVID-19 patients for reliable, interpretable, and universal mortality model evaluation. Through multiple analyses including RF analysis, association analysis, time-series analysis, etc., the mortality prediction model was established, evaluated, and achieved clinically creditable prediction power on the Wuhan cohort and Würzburg cohort.

The mortality prediction model proposed in this study could help identify critically ill patients early and provide preferential treatment for each individual. Firstly, the five important clinical features (lymphocyte (%), neutrophil count, CRP, LDH, and α-HBDH) were identified. These five features could reflect several important aspects of disease development, such as viral infection (Trowell, 1947), coexistence of other infections (Xie et al., 2020), immune reaction during pneumonia (Sabrina et al., 2012), the severity of inflammatory response (Vermeire et al., 2004), tissue/cell damage, and cardiac injury (Sanwald and Kirk, 1966; Kishaba et al., 2014), which could provide more information to monitor the progression of patients. Secondly, these five features could be used for predicting the outcome of COVID-19 patients with high accuracy. Thirdly, the foresight of the mortality prediction model was strong up to as early as 40 days or more before discharge. This indicates that our model could allow resource optimization to be conducted many days ahead, and physicians can make a preliminary judgment on the prognosis of patients according to this model to prompt the choice of clinical intervention in later stages.

Our mortality prediction model shows superior prediction power at different time points during the course of the disease. Robust prediction power at different time points (Figure 4) also suggests that the mortality prediction model provides important indicators for disease monitoring, indicating early clinical intervention for clinical treatment. Our mortality prediction model also shows superior prediction power for different preexisting diseases of patients, indicating the robustness of the mortality prediction model. These results could serve well as the basis for personalized treatment of COVID-19 patients.

Our finding in the Wuhan cohort (model development) has also been tested in an independent cohort from Germany (Würzburg cohort). Although the international aspects such as the ethnicities, healthcare systems, hygienic measures, local regulations, and management strategies, as well as their average age (*t*-test, *p* = 0.0005), are different in these two cohorts, our mortality prediction model has also shown the high prediction power in tens of days ahead of patients’ discharge, underlining the robustness and the foresight of this model.

The second COVID-19 wave in Europe is ongoing. This mortality prediction model has been validated at a European center and might provide a useful instrument for triaging the patients and optimizing the resources. Because we have a series of mortality prediction models with constant high accuracy along with the whole duration of patients’ stay in the hospital, we could adjust treatment for possibly serious patients on a day-to-day basis to reduce the mortality rate of patients with COVID-19 as much as possible. In addition, our study also provides new insight into the mortality prediction model’s application value in other infectious disease outbreaks in the future.

In conclusion, this study has established a mortality prediction model that allowed early classification of COVID-19 towards personalized treatment in these patients, not only at admission but also along the treatment timeline. This model may represent a valuable tool for triaging and optimizing the resources in patients with COVID-19 infection worldwide.

## Data Availability

The raw data used in this study are not publicly available due to the confidential policy of National Health Commission of China, as well as the General Data Protection Regulation (GDPR) of European Union (EU), but are available from the corresponding author Xiaohua Hou upon reasonable request.
