# Supplementary material for: Reliable and Interpretable Mortality Prediction With Strong Foresight in COVID-19 Patients: An International Study From China and Germany
Source: Front Artif Intell. 2021 Sep 3;4:672050. doi: 10.3389/frai.2021.672050 (PMC8446629; doi:10.3389/frai.2021.672050)
Supplement: Supplementary file 1 [file DataSheet1.PDF]

Reliable and interpretable mortality prediction with strong foresight in COVID-19 patients: an international study from China and Germany

Tao Bai[a,1], Xue Zhu[a,2], Xiang Zhou[3], Denise Grathwohl[3], Pengshuo Yang[2], Yuguo Zha[2], Yu Jin[1], Hui Chong[2], Qingyang Yu[2], Nora Isberner[3], Dongke Wang[1], Lei Zhang[1], K Martin Kortüm[3], Jun Song[1], Leo Rasche[3], Hermann Einsele[3], Kang Ning[*,2], Xiaohua Hou[*,1]

[1]Division of Gastroenterology, Union Hospital, Tongji Medical College, Huazhong University of Science and Technology, Wuhan 430022, China

[2]Key Laboratory of Molecular Biophysics of the Ministry of Education, Hubei Key Laboratory of Bioinformatics and Molecular-imaging, Department of Bioinformatics and Systems Biology, College of Life Science and Technology, Huazhong University of Science and Technology, Wuhan 430074, China

[3]Department of Internal Medicine II, University Hospital of Würzburg, 97080 Würzburg, Germany

[a]Tao Bai and Xue Zhu contributed equally to this study

[*]Corresponding author:

Kang Ning, PhD, E-mail: ningkang@hust.edu.cn

Xiaohua Hou, PhD., MD, E-mail: houxh@hust.edu.cn

Supplementary Figures and Tables

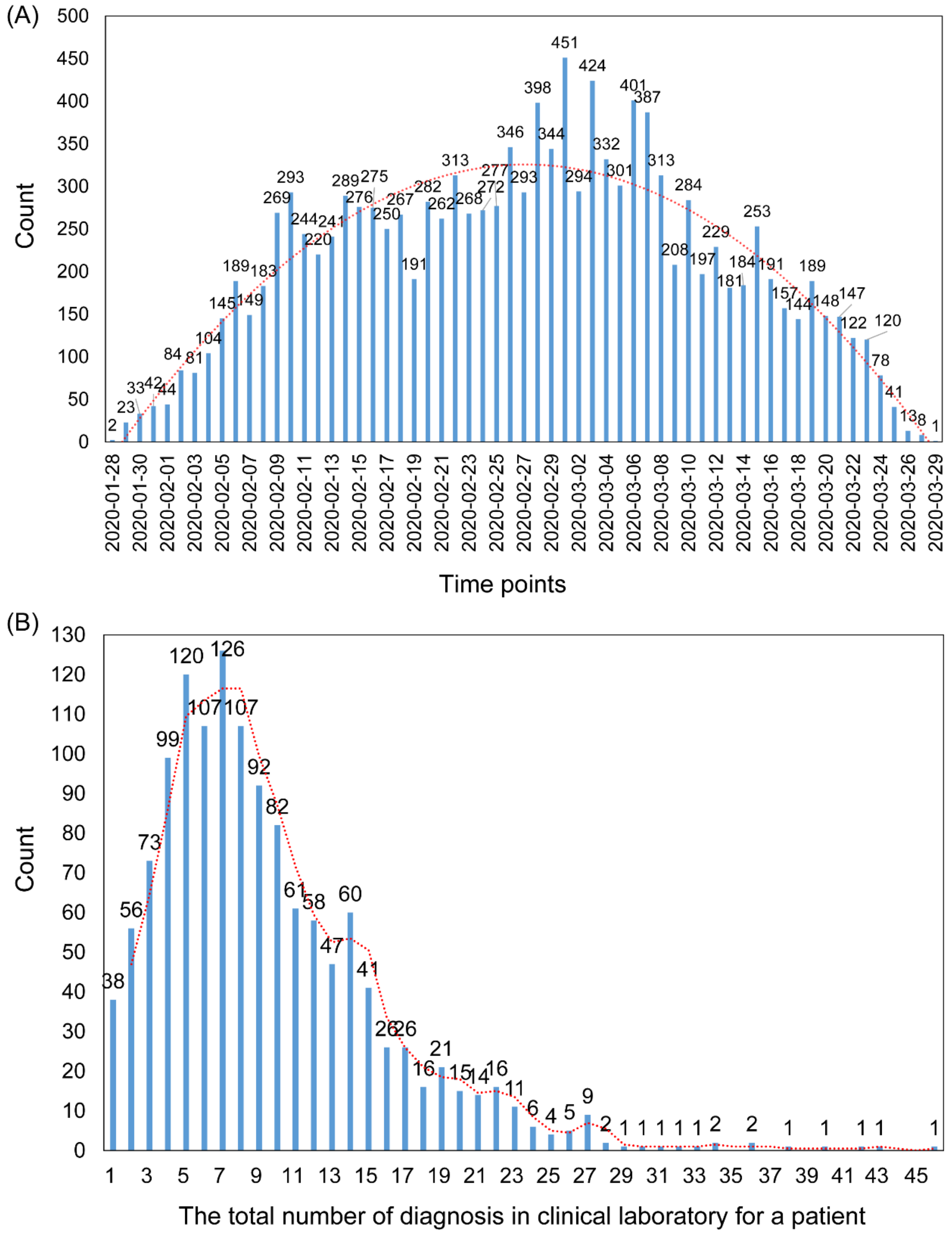

Supplementary Figure 1. The frequency distribution of COVID-19 patients with time points and the total number of clinical diagnoses. (A), The distribution of the count number of examined COVID-19 patients in clinical laboratory daily from 28

January 2020 to 29 March 2020. (B), The frequency distribution of the number of tests in clinical laboratory for COVID-19 patients. Here, the y-axis represents the number of samples or patients.

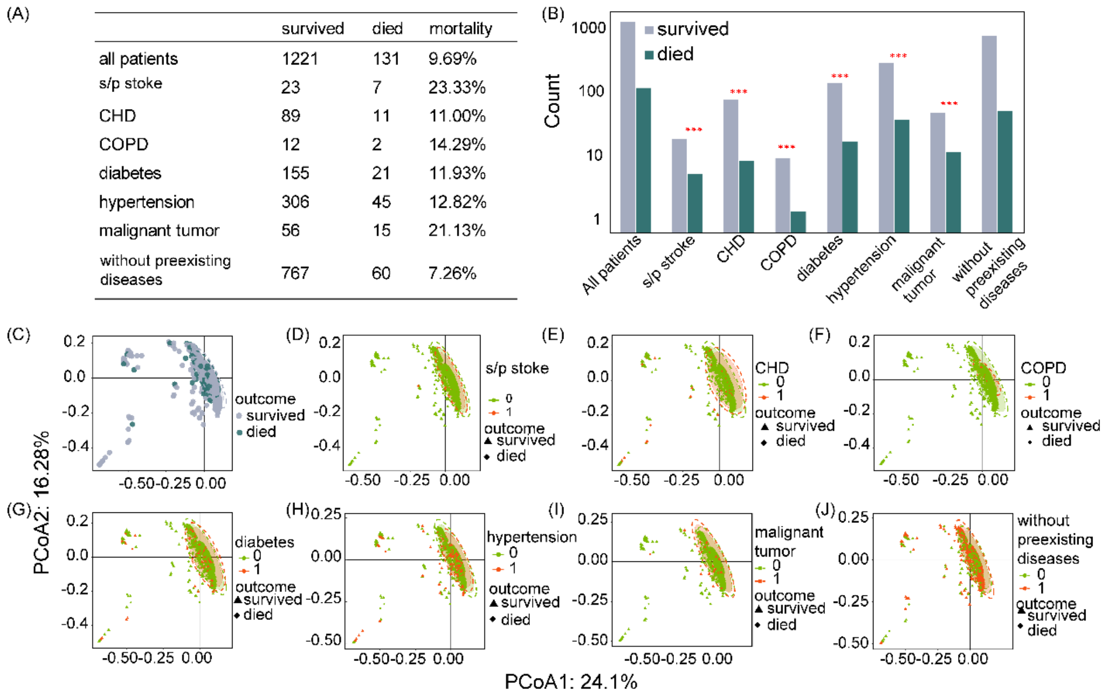

	survived	died	mortality
all patients	1221	131	9.69%
s/p stoke	23	7	23.33%
CHD	89	11	11.00%
COPD	12	2	14.29%
diabetes	155	21	11.93%
hypertension	306	45	12.82%
malignant tumor	56	15	21.13%
without preexisting diseases	767	60	7.26%

Supplementary Figure 2. Distribution of 1,352 samples based on 101 numerical features. (A) Number of COVID-19 patients with or without different types of preexisting diseases. (B) Distribution of COVID-19 patients with or without different types of preexisting diseases. PCoA analysis using Jaccard coefficient for distance measurement based on all samples grouped by (C) the outcome of COVID-19 patients, the preexisting diseases: (D) s/p stroke, (E) CHD, (F) COPD, (G) diabetes, (H) hypertension, (I) malignant tumor, and (J) without preexisting diseases. In D-J, the orange color (marked as "1") indicates that the patient has this type of preexisting disease, while the light green (marked as "0") indicates they do not have. The triangle represents survived patients, while the rhombus represents expired patients. The 90% confidence intervals for each group are also shown in the background.

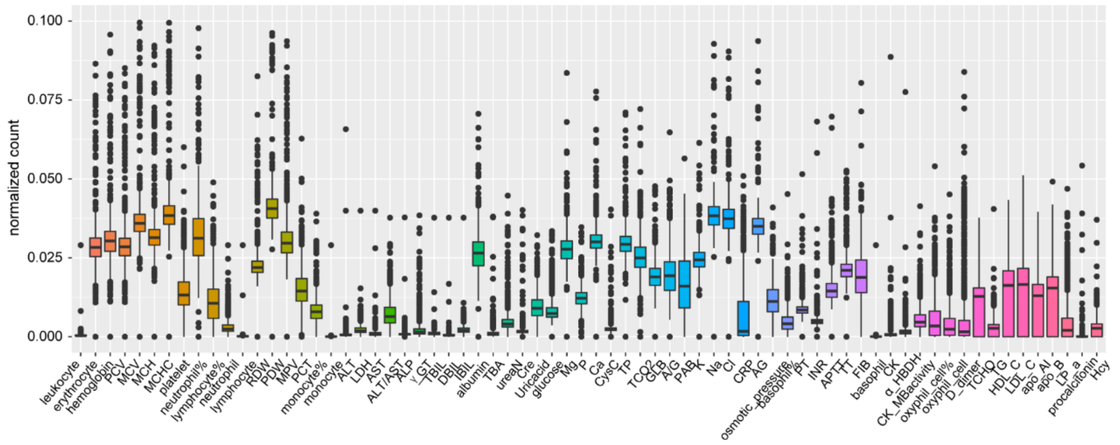

Supplementary Figure 3. The distribution of each feature with an average abundance no less than 0.001 based on Wuhan cohort. Based on the normalized abundance for these clinical features, these features are identified with a different distribution.

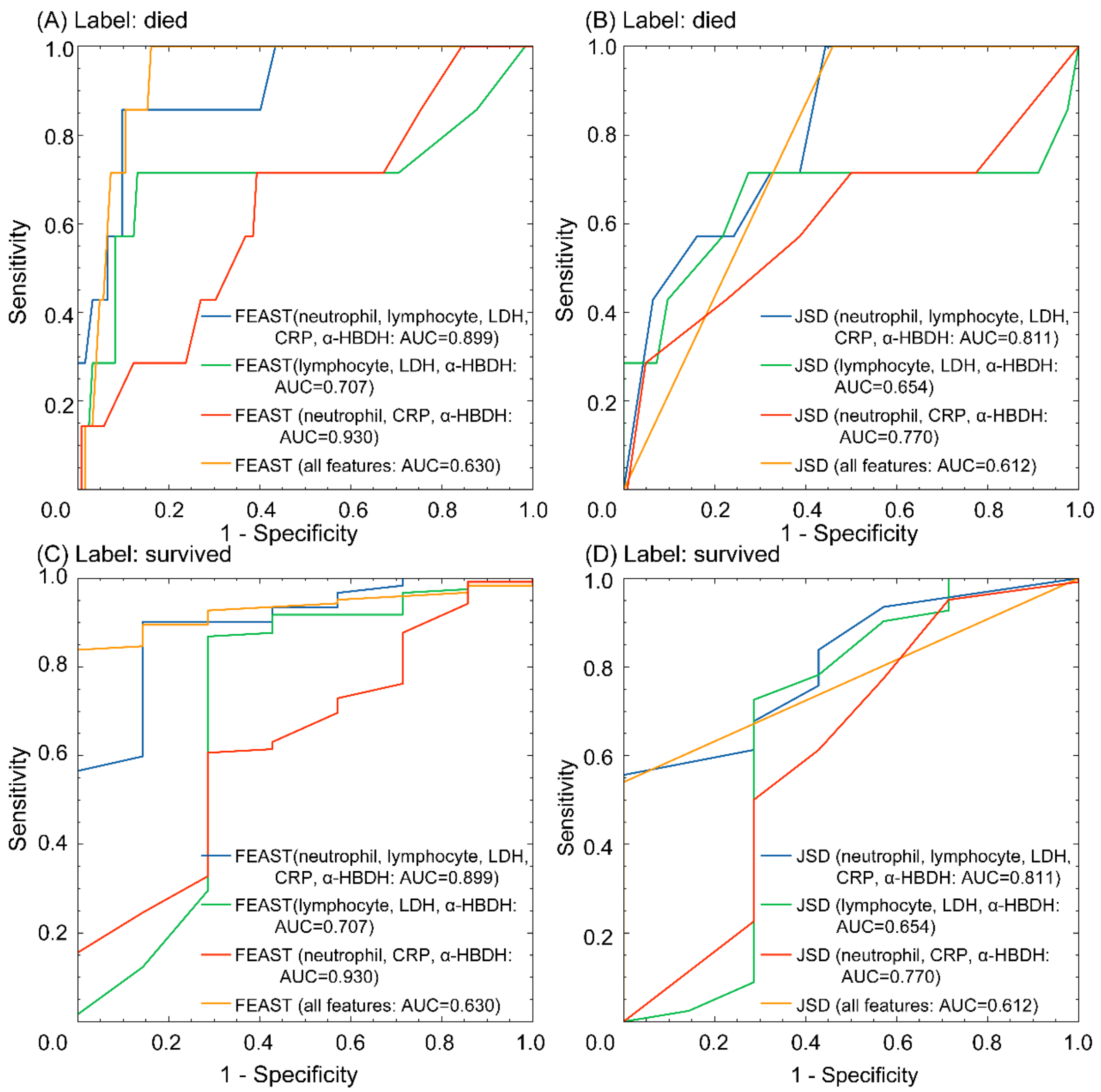

Supplementary Figure 4. The prediction result of the outcome of COVID-19 patients from Wuhan cohort using FEAST and JSD based on all clinical features, top five clinical features and different combinations of the subset of these five clinical features. The prediction result based on FEAST (A) & (C), JSD (B) & (D). The result also showed the best distinguishing power when using top five features to construct the model.

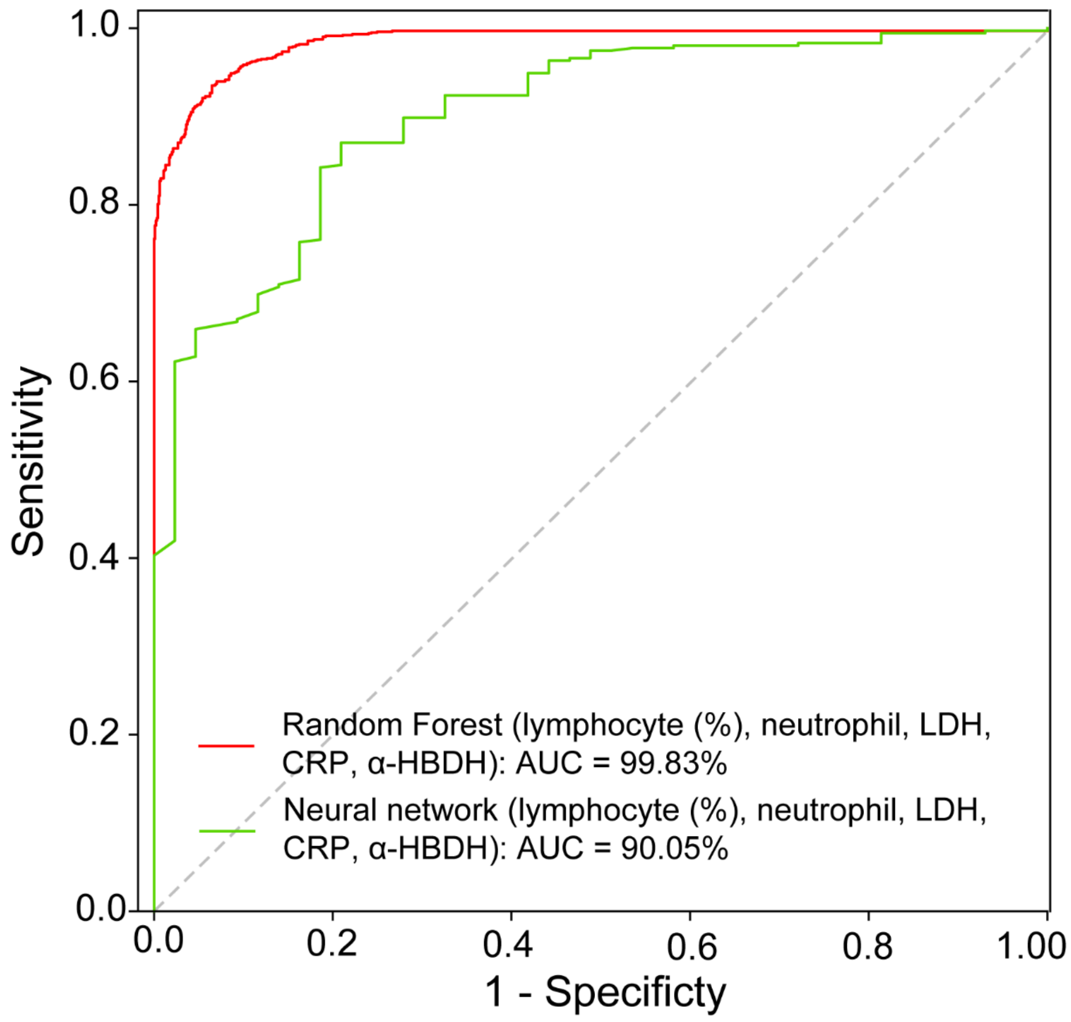

	Neural network	Random Forest
Precision	0.9136	0.9700
Recall or Sensitivity	0.9831	0.9100
Specifictiy	0.2326	0.9600
F1 score	0.9471	0.9400
Accuracy	0.9186±0.0083	0.9142±0.1700
ROC	0.9005	0.9983

Supplementary Figure 5. Comparison of the performance between Random Forest model and neural network in predicting the outcome of COVID-19 patients. This comparison was based on the combination of five best distinguishing clinical features: lymphocyte (%), neutrophil, LDH, CRP, α-HBDH collected from Wuhan cohort at admission. The result also showed the prediction power of Random Forest model in predicting the COVID-19 patients' outcome.

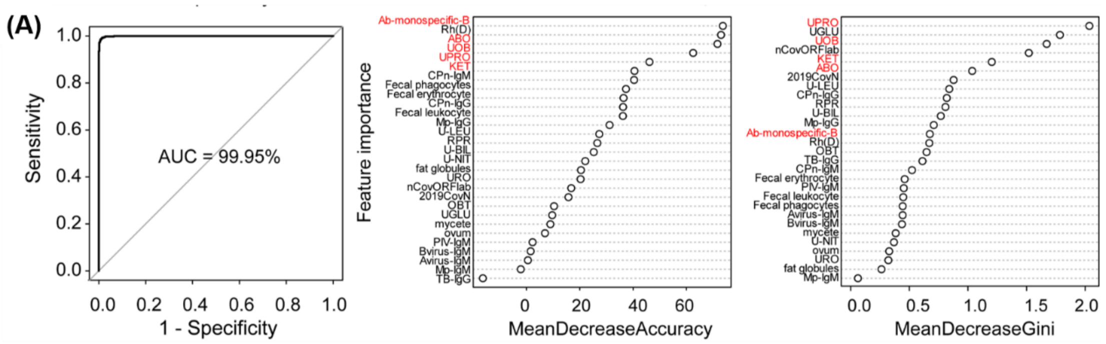

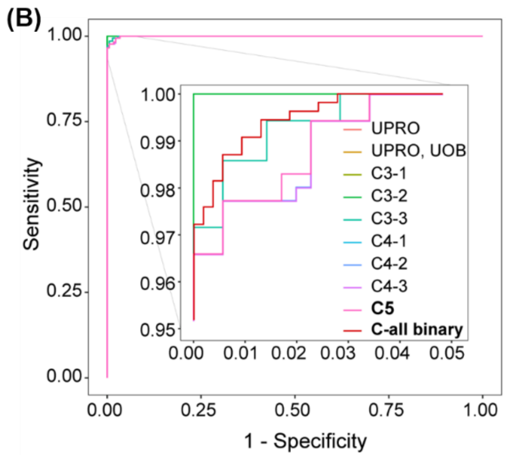

For curves	Variable	Precision	Recall or Sensitivity	Specificity	F1 score	Accurary(%)	AUC(%)
**UPRO**	**UPRO**	**1.00**	**0.96**	**1.00**	**1.00**	**92.33±0.02**	**100.00**
UPRO, UOB	UPRO, UOB	1.00	0.96	1.00	1.00	92.33±0.04	100.00
C3-1	UPRO, UOB, Ab-monospe-B	1.00	0.96	0.99	0.98	91.76±0.110	99.94
C3-2	UPRO, UOB, ABO	1.00	0.96	1.00	1.00	92.33±0.02	100.00
**C3-3**	**UPRO, UOB, KET**	**1.00**	**0.96**	**0.98**	**1.00**	**92.61±0.06**	**99.96**
C4-1	UPRO, UOB, ABO, KET	1.00	0.99	0.98	1.00	91.76±0.107	99.94
C4-2	UPRO, UOB, ABO, Ab-monospe-B	1.00	0.98	0.99	0.99	91.76±0.108	99.94
C4-3	UPRO, UOB, KET, Ab-monospe-B	1.00	0.98	0.99	0.99	91.76±0.109	99.94
**C5**	**UPRO, UOB, ABO, KET, Ab-monospe-B**	**1.00**	**0.96**	**0.98**	**0.98**	**92.10±0.22**	**99.94**
**C-all binary**	**All binary features**	**0.95**	**0.94**	**0.94**	**0.94**	**91.66±0.16**	**99.95**

Supplementary Figure 6. The clinical feature selection and mortality prediction results utilizing the first samples from Wuhan cohort at admission. (A), Clinical feature selection based on 625 samples and 29 binary features. These features were ranked by MeanDecreasedAccuracy and MeanDecreasedGini according to their importance. Considering both of these two import Random Forest parameters, we have selected five important features: UPRO, UOB, ABO, KET and Ab-monospecific-B(blood). (B), Comparison of ROC curves and diagnostic performances of all clinical features, sub-features' combinations according to their importance using the first samples at admission (also referred to as admission-day0). Among all combinations, the combination of UPRO, UOB and KET (accuracy: 99.61%, AUC: 99.96%) was outstanding from the others, followed by UPRO, all binary features and the combination of these five features. However, since binary clinical features are available

for a maximum of 46.2% patients (625 patients at admission), which is relatively incomplete for systematic evaluation, we left the analyses on these binary features for subsequent examination in larger cohorts of patients in the future.

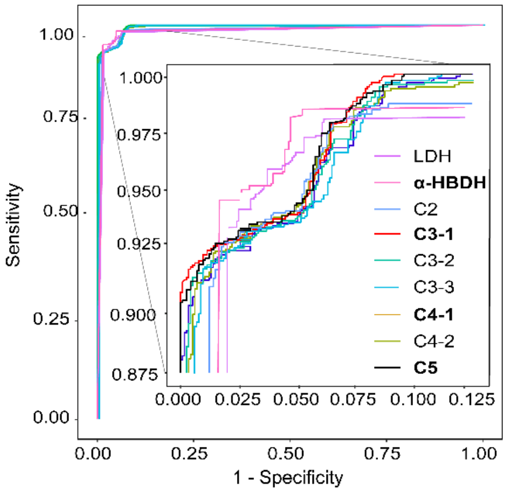

For curves	Variables	Precision	Recall or Sensitivity	Specificity	F1 score	Accurary(%)	AUC(%)
LDH	LDH	0.98	0.91	0.91	0.94	92.59±0.06	97.99
**α-HBDH**	**α-HBDH**	**0.98**	**0.95**	**0.98**	**0.97**	**92.95±0.02**	**98.36**
C2	LDH, α-HBDH	0.99	0.98	0.93	0.99	93.21±0.06	98.52
**C3-1**	**Neutrophil, LDH, α-HBDH**	**0.97**	**1.00**	**0.93**	**0.98**	**93.76±0.07**	**99.60**
C3-2	Lymphocyte(%), LDH, α-HBDH	1.00	1.00	0.99	1.00	93.74±0.06	99.32
C3-3	LDH, CRP, α-HBDH	0.99	1.00	0.92	0.99	93.60±0.08	99.59
**C4-1**	**Neutrophil, LDH, CRP, α-HBDH**	**0.97**	**0.99**	**0.93**	**0.98**	**94.04±0.08**	**99.54**
C4-2	Lymphocyte(%), neutrophil, LDH, α-HBDH	0.99	0.99	0.93	0.99	93.68±0.08	99.47
**C5**	**Lymphocyte**(%), **neutrophil, LDH, CRP, α-HBDH**	**0.98**	**0.99**	**0.93**	**0.98**	**94.03±0.11**	**99.60**

Supplementary Figure 7. Comparison of the diagnostic performances of the five clinical features and their sub-features' combinations according to their importance using the final samples from Wuhan cohort at discharge. Here, the time point of the final samples at discharge was also referred to as discharge-day0.

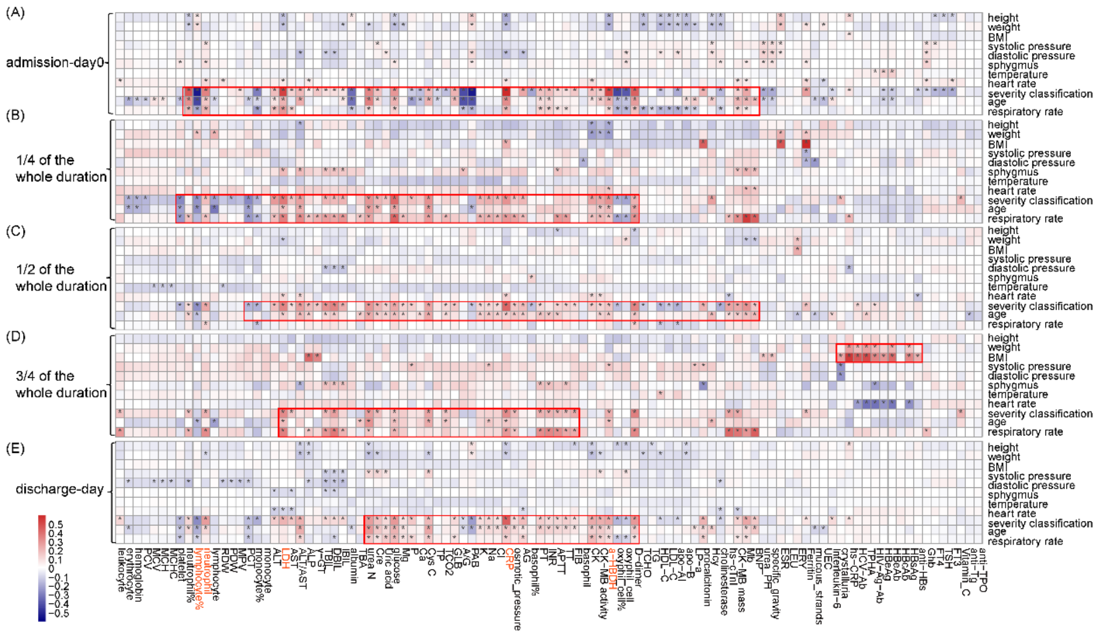

Supplementary Figure 8. Associations between clinical features and phenotypic characteristics based on data of the time point corresponding to 0, 1/4, 1/2, 3/4 and 1 of the whole duration in the hospital for Wuhan cohort. (A) Based on data of the time point that corresponds to 0 of the whole duration in the hospital (also referred to admission-day0). (B) Based on data of the time point that corresponds to 1/4 of the whole duration in the hospital. (C) Based on data of the time point that corresponds to 1/2 of the whole duration in the hospital. (D) Based on data of the time point that corresponds to 3/4 of the whole duration in the hospital. (E) Based on data of the time point that corresponds to 1 of the whole duration in the hospital (also referred to discharge-day). Note: *represents a significant correlation between a phenotypic characteristic and a clinical feature (Pearson correlation $P < 0.05$).

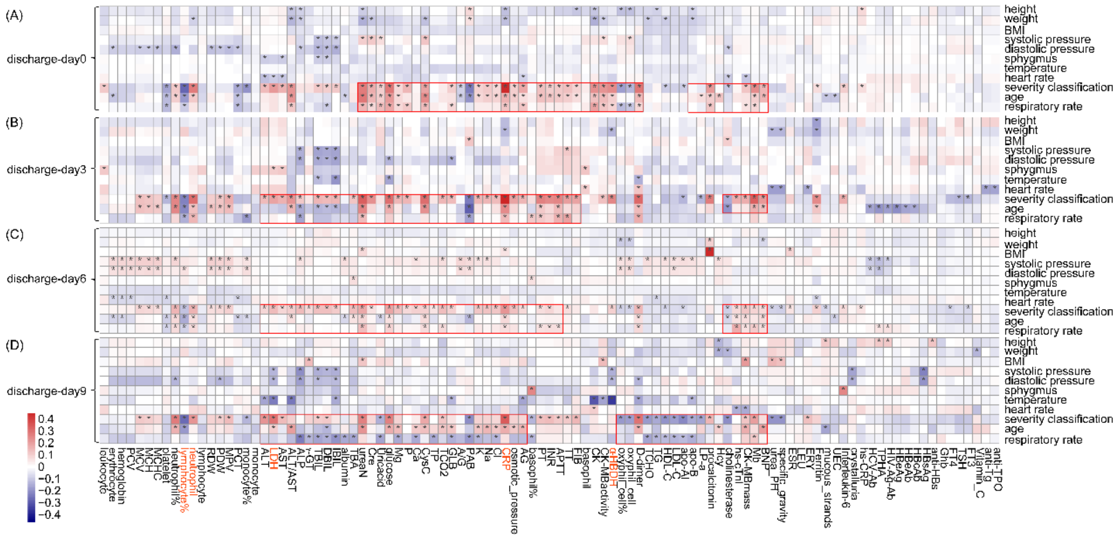

Supplementary Figure 9. Associations of clinical features and phenotypical characteristics based on several representative time points for Wuhan cohort. (A), Based on the discharge day (discharge-day0); (B), Based on the third day to discharge (discharge-day3); (C), Based on the sixth day to discharge (discharge-day6); (D), Based on the ninth day to discharge (discharge-day9). Here, the orange color marked clinical features are the five selected clinical features, except for lymphocyte (%), they are significant and positive correlation with age, respiratory rate and severity classification of patients along the time-series, while the blue color marked clinical features were significant with age, respiratory rate and severity classification of patients along the time-series. Note: *represents a significant correlation between a phenotypic characteristic and a clinical feature (Pearson correlation: $P < 0.05$).

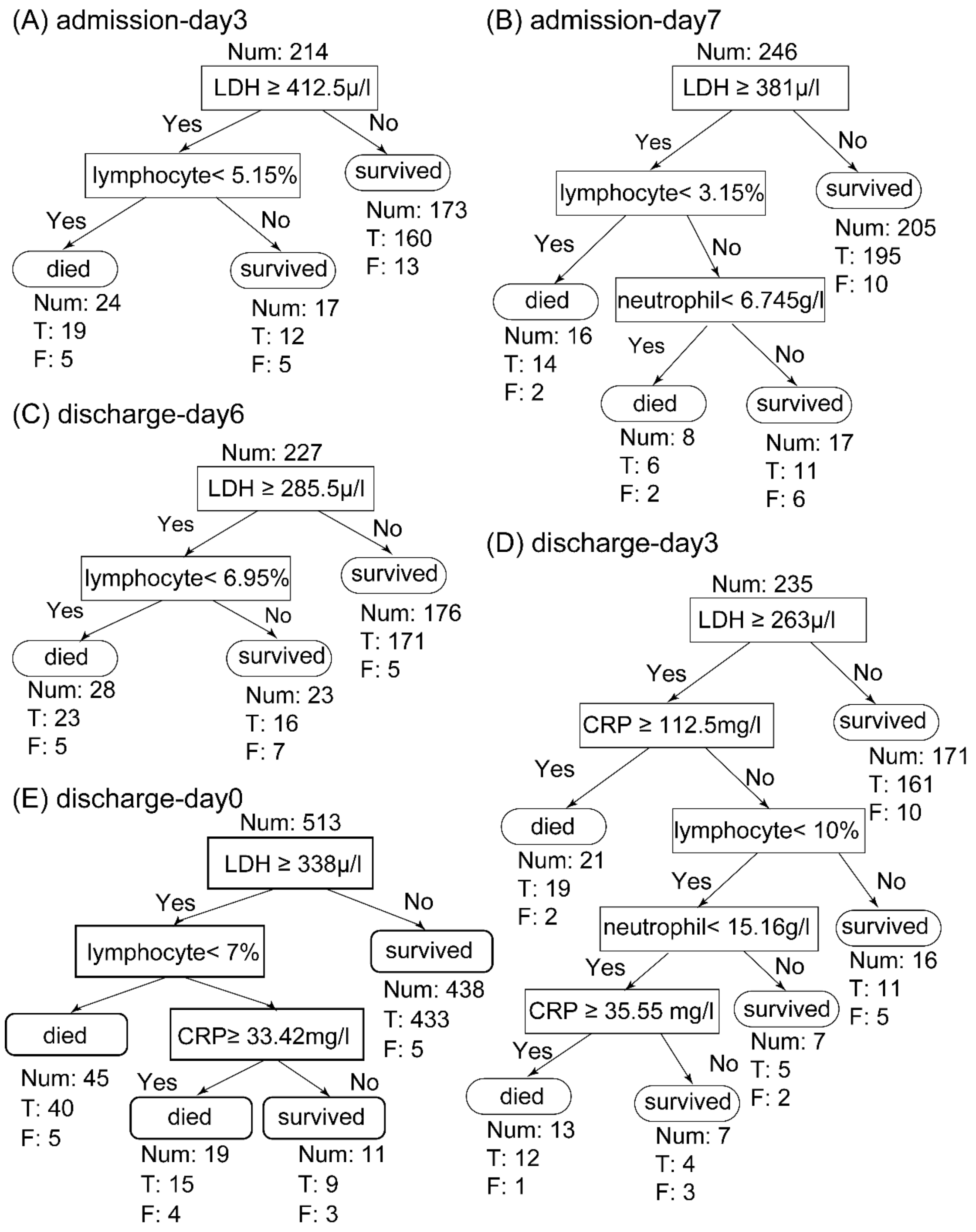

Supplementary Figure 10. The decision tree for prediction the outcome of COVID-19 patients using clinical features based on the Wuhan cohort. (A), Based on the data of admission-day3. (B), Based on the data of admission-day7. (C), Based on the data of discharge-day6. (D), Based on the data of discharge-day3. (E), Based on the data of discharge-day0.The dynamic decision trees were built based on several time

points for assisting the clinical diagnosis for COVID-19 patients. Annotations: Num: The number of patients in predictor. T: The number of correctly matched patients. F: The number of mismatched patients. Here, the Num above the root was the total samples used for building the binary decision tree (also referred to as the number of samples for training).

(A)

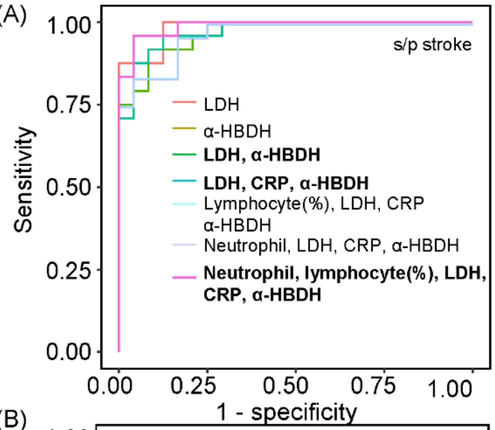

Variables	Precision	Recall or Sensitivity	Specificity	F1 score	Accurary(%)	AUC(%)
LDH	0.67	1.00	0.88	0.80	87.52±0.11	98.44
α-HBDH	1.00	0.96	0.88	0.98	91.65±0.52	96.53
**LDH, α-HBDH**	**1.00**	**0.92**	**0.92**	**0.96**	**91.66±0.21**	**96.88**
**LDH, CRP, α-HBDH,**	**1.00**	**0.88**	**0.96**	**0.93**	**92.36±1.6**	**97.22**
Lymphocyte(%) LDH, CRP, α-HBDH	1.00	0.96	0.88	0.98	91.59±0.69	96.53
Neutrophil, LDH, CRP, α-HBDH	1.00	0.96	0.83	0.98	91.65±0.32	96.53
**Neutrophil, lymphocyte(%), LDH, CRP, α-HBDH**	**1.00**	**0.96**	**0.96**	**0.98**	**87.42±0.59**	**98.78**

(B)

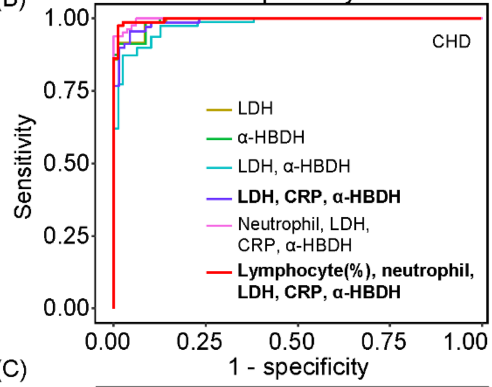

Variables	Precision	Recall or Sensitivity	Specificity	F1 score	Accurary(%)	AUC(%)
LDH	0.93	0.92	1.00	0.92	85.74±0.42	99.16
α-HBDH	0.94	0.91	0.99	0.93	85.01±0.33	99.14
LDH,α-HBDH	1.00	0.87	0.98	0.93	90.90±0.10	97.76
LDH, CRP, α-HBDH	1.00	0.98	0.98	0.99	91.16±0.58	98.97
Neutrophil, LDH, CRP, α-HBDH	1.00	0.94	1.00	0.97	89.02±0.20	98.97
Lymphocyte(%), neutrophil, LDH, CRP, α-HBDH	0.93	0.99	0.98	0.96	96.13±0.41	99.66

(C)

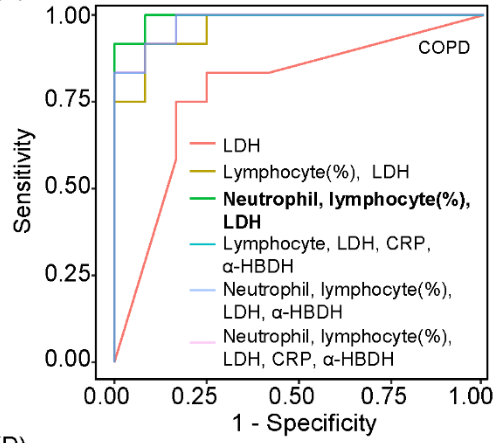

Variables	Precision	Recall or Sensitivity	Specificity	F1 score	Accurary(%)	AUC(%)
LDH	1.00	0.83	0.75	0.91	74.50±0.05	78.74
**Lymphocyte(%), LDH**	1.00	0.92	0.92	0.96	75.03±0.52	96.53
**Neutrophil, lymphocyte(%), LDH**	**1.00**	**1.00**	**0.92**	1.00	**75.01±0.37**	**99.31**
Lymphocyte(%), LDH, CRP, α-HBDH	1.00	1.00	0.83	**1.00**	74.99±0.47	97.92
Neutrophil, lymphocyte(%), LDH, α-HBDH	1.00	1.00	0.83	1.00	75.00±0.21	97.92
Neutrophil, lymphocyte(%), LDH, CRP, α-HBDH	1.00	1.00	0.83	1.00	74.69±1.58	97.92

(D)

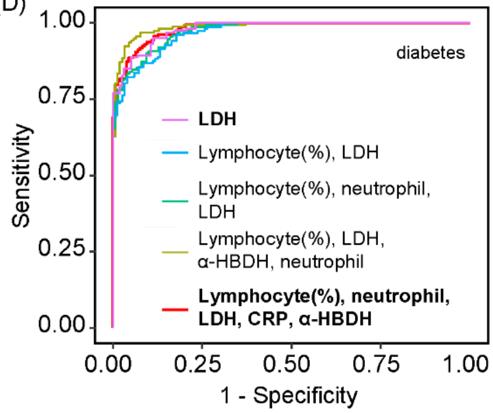

Variable	Precision	Recall or Sensitivity	Specificity	F1 score	Accurary(%)	AUC(%)
**LDH**	**0.92**	**0.95**	**0.89**	**0.94**	**91.57±0.84**	**98.01**
Lymphocyte(%), LDH	0.95	0.93	0.86	0.94	86.05±0.79	96.97
Lymphocyte(%), neutrophil, LDH	0.79	0.91	0.90	0.85	89.42±0.55	97.41
Lymphocyte(%), neutrophil, LDH, α-HBDH	0.79	0.97	0.92	0.87	87.03±0.50	98.72
**Lymphocyte(%), neutrophil, LDH, CRP, α-HBDH**	**0.94**	**0.96**	**0.89**	**0.95**	**88.69±0.85**	**98.17**

(E)

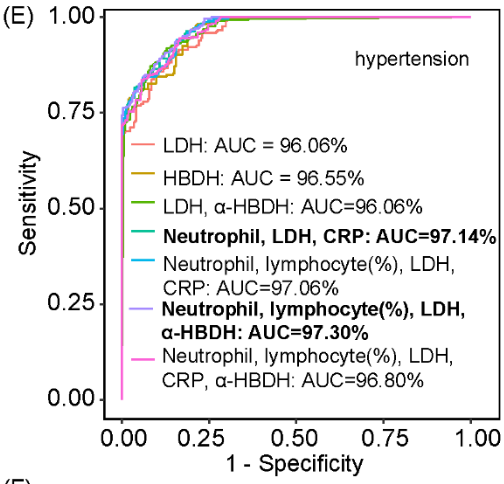

Variable	Precision	Recall or Sensitivity	Specificity	F1 score	Accurary(%)	AUC(%)
LDH	0.94	0.91	0.85	0.92	86.69±0.33	96.06
α- HBDH	0.94	0.96	0.81	0.95	82.91±0.35	96.55
LDH, α-HBDH	0.97	0.92	0.87	0.94	86.28±0.22	96.53
**Neutrophil, LDH, CRP**	**0.92**	**0.89**	**0.89**	**0.90**	**90.22±0.23**	**97.14**
Neutrophil, lymphocyte(%), LDH, CRP	0.92	0.94	0.84	0.93	89.70±0.27	97.06
**Neutrophil, lymphocyte(%), LDH, α- HBDH**	**0.92**	**0.94**	**0.85**	**0.93**	**89.48±0.16**	**97.30**
Neutrophil, lymphocyte(%), LDH, CRP, α- HBDH	1.00	0.97	0.93	0.98	89.39±0.19	96.80

(F)

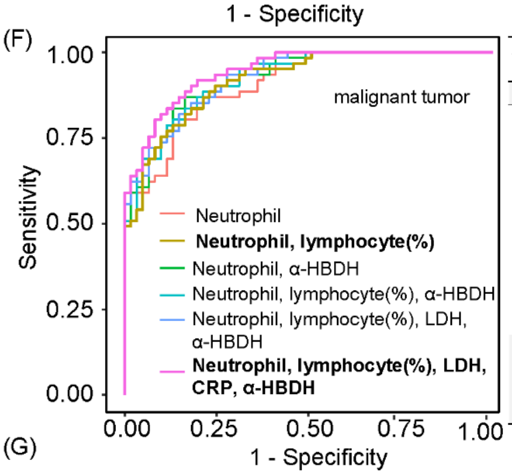

Variable	Precision	Recall or Sensitivity	Specificity	F1 score	Accurary(%)	AUC(%)
**Neutrophil**	**0.71**	**0.87**	**0.80**	**0.78**	**80.33±0.11**	**91.19**
Neutrophil, lymphocyte(%)	1.00	0.84	0.82	0.91	81.98±0.21	91.91
Neutrophil, α-HBDH	0.86	0.84	0.82	0.85	83.65±0.39	92.42
Neutrophil, lymphocyte(%), α-HBDH	0.86	0.89	0.79	0.87	83.60±0.19	92.48
Neutrophil, lymphocyte(%), LDH, α-HBDH	1.00	0.85	0.82	0.92	81.96±0.20	92.72
**Neutrophil, lymphocyte(%), LDH, CRP, α-HBDH**	**1.00**	**0.92**	**0.80**	**0.96**	**84.72±0.78**	**94.54**

(G)

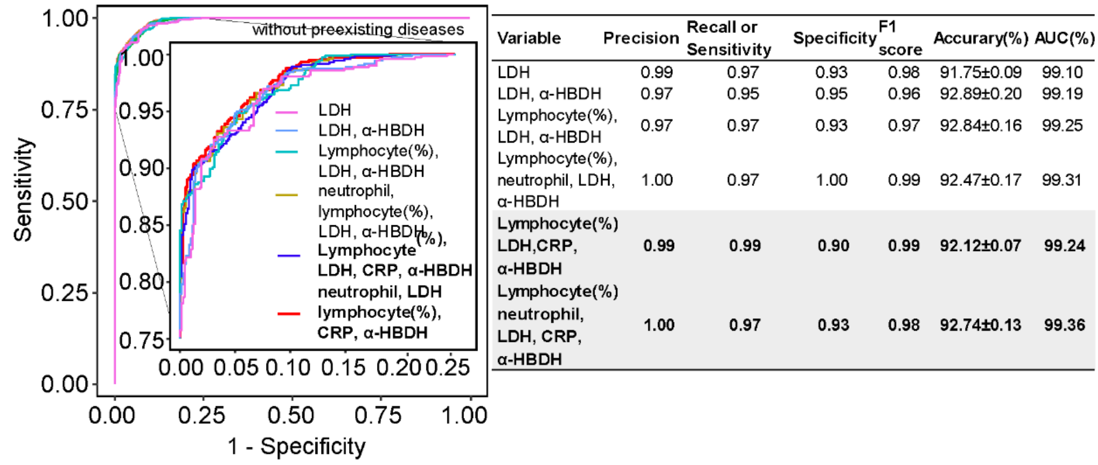

Variable	Precision	Recall or Sensitivity	Specificity	F1 score	Accurary(%)	AUC(%)
LDH	0.99	0.97	0.93	0.98	91.75±0.09	99.10
LDH, α-HBDH	0.97	0.95	0.95	0.96	92.89±0.20	99.19
Lymphocyte(%), LDH, α-HBDH	0.97	0.97	0.93	0.97	92.84±0.16	99.25
Lymphocyte(%), neutrophil, LDH, α-HBDH	1.00	0.97	1.00	0.99	92.47±0.17	99.31
**Lymphocyte(%) LDH,CRP, α-HBDH**	**0.99**	**0.99**	**0.90**	**0.99**	**92.12±0.07**	**99.24**
**Lymphocyte(%) neutrophil, LDH, CRP, α-HBDH**	**1.00**	**0.97**	**0.93**	**0.98**	**92.74±0.13**	**99.36**

Supplementary Figure 11. Comparison of diagnostic performances of lymphocyte (%), neutrophil count, LDH, CRP, α-HBDH, and their combinations in discriminating the outcome of COVID-19 patients based on different kinds of preexisting diseases. (A) Based on s/p stroke dataset. (B) Based on CHD dataset. (C) Based on COPD dataset. (D) Based on diabetes dataset. (E) Based on hypertension

dataset. (F) Based on malignant tumor dataset. (G) Based on patients without preexisting diseases.

Supplementary Table 1. An overview of COVID-19 patients from the Wuhan cohort in this study. Among all of the 1352 patients, fever (74.56%) was the most pervasive clinical symptom, followed by cough (60.43%), fatigue (47.86%), diarrhea (41.68%), chest distress (34.10%), dyspnoea (31.66%) and muscular soreness (24.63%).

	Feature	Overall	Proportion (%)
**Gender**	Male	683	50.22
	Female	669	49.48
**Age**	Age	58.22±14.90	
**Epidemiological history**	Wuhan residents	1,352	100
	Birthplace (Hubei)	1,183	87.5
	Birthplace (Wuhan)	1,019	75.37
	Retiree	454	33.58
**Clinical symptoms**	Fever	1,008	75.46
	Cough	817	60.43
	Fatigue	647	47.96
	Diarrhea	566	41.68
	Chest distress	461	34.1
	Dyspnoea	428	31.66
	Muscular soreness	333	24.63
	Stomachache	196	14.5
	Dizziness	99	7.3

	Headache	18	1.3
**Outcome**	Survived	1,221	90.31
	Died	131	9.67

Supplementary Table 2. An overview of COVID-19 patients from the Würzburg cohort. Among all of the 81 patients, respiratory symptoms were the most common clinical symptom, 53.67% of them have respiratory failure.

	**Feature**	**Overall**	**Proportion (%)**
**Gender**	Male	51	62.96
	Female	30	37.04
**Age**	Age	67.15±15.17	
**Clinical symptoms**	Respiratory symptoms	61	74.39
	Respiratory failure	44	53.67
	Shock	22	26.83
	Mechanical ventilation	34	41.46
**Outcome**	Survived	72	88.89
	Died	9	11.11
